# Alterations in Gut Microbiota Composition Are Associated with Changes in Emotional Distress in Children with Obstructive Sleep Apnea

**DOI:** 10.3390/microorganisms12122626

**Published:** 2024-12-18

**Authors:** Chung-Guei Huang, Wan-Ni Lin, Li-Jen Hsin, Yu-Shu Huang, Li-Pang Chuang, Tuan-Jen Fang, Hsueh-Yu Li, Terry B. J. Kuo, Cheryl C. H. Yang, Chin-Chia Lee, Li-Ang Lee

**Affiliations:** 1 Department of Laboratory Medicine, Linkou Main Branch, Chang Gung Memorial Hospital, Taoyuan 33305, Taiwan; joyce@cgmh.org.tw; 2 Research Center for Emerging Viral Infections, Department of Medical Biotechnology and Laboratory Science, Chang Gung University, Taoyuan 33302, Taiwan; 3 Department of Otorhinolaryngology-Head and Neck Surgery, Linkou Main Branch, Chang Gung Memorial Hospital, Chang Gung University, Taoyuan 33305, Taiwan; wannilin@hotmail.com (W.-N.L.); lijen.hsin@gmail.com (L.-J.H.); fang3109@cgmh.org.tw (T.-J.F.); hyli38@cgmh.org.tw (H.-Y.L.); 4 School of Medicine, College of Medicine, Chang Gung University, Taoyuan 33302, Taiwan; yushuhuang1212@gmail.com (Y.-S.H.); r5243@cgmh.org.tw (L.-P.C.); meiluosha@gmail.com (C.-C.L.); 5 Department of Child Pschiatry, Linkou Main Branch, Chang Gung Memorial Hospital, Chang Gung University, Taoyuan 33305, Taiwan; 6 Department of Pulmonary and Critical Care Medicine, Linkou Main Branch, Chang Gung Memorial Hospital, Taoyuan 33305, Taiwan; 7 Institute of Brain Science, National Yang Ming Chiao Tung University, Taipei 112304, Taiwan; tbjkuo@nycu.edu.tw (T.B.J.K.); cchyang@ym.edu.tw (C.C.H.Y.); 8 Sleep Research Center, National Yang Ming Chiao Tung University, Taipei 112304, Taiwan; 9 Center for Mind and Brain Medicine, Tsaotun Psychiatric Center, Ministry of Health and Welfare, Nantou 542019, Taiwan; 10 School of Medicine, College of Life Science and Medicine, National Tsing Hua University, Hsinchu 300044, Taiwan

**Keywords:** gut microbiome, obstructive sleep apnea, emotional distress, dietary profile, sleep heart rate, children, adenotonsillectomy, educational counseling

## Abstract

Emerging evidence underscores the pivotal role of the gut microbiota in regulating emotional and behavioral responses via the microbiota–gut–brain axis. This study explores associations between pediatric obstructive sleep apnea (OSA), emotional distress (ED), and gut microbiome alterations before and after OSA treatment. Sixty-six children diagnosed with OSA via polysomnography participated, undergoing adenotonsillectomy alongside routine educational sessions. ED was assessed using the OSA-18 questionnaire, categorizing participants into high ED (scores ≥ 11, 52%) and low ED (scores < 11, 48%) groups. Gut microbiome analysis revealed significant diversity differences, with high ED linked to a reduced Shannon index (*p* = 0.03) and increased beta diversity (*p* = 0.01). Three months post-treatment, significant improvements were observed in OSA symptoms, ED scores, and gut microbiome alpha diversity metrics among 55 participants (all *p* < 0.04). Moreover, changes in the relative abundances of *Veillonella*, *Bifidobacterium*, *Flavonifractor*, and *Agathobacter*, as well as ultra-low frequency power and low frequency power of sleep heart rate variability, were independently associated with ED score alterations. These findings underscore the gut microbiome’s critical role in the emotional and behavioral symptoms associated with pediatric OSA, suggesting that microbiome-targeted interventions could complement traditional treatments for ED reduction and emphasizing the need for further research.

## 1. Introduction

Obstructive sleep apnea (OSA) has emerged as a pressing pediatric health concern, affecting approximately 1.2% of children globally [1]. The growing prevalence of OSA over the last decade underscores the need for a deeper understanding of its underlying mechanisms and more effective interventions [2]. OSA not only disrupts sleep but also negatively impacts cognitive, behavioral, and physiological functions in children [3], manifesting in behavioral and emotional distress (ED) such as hyperactivity, attention deficits, and emotional regulation problems [4,5]. These disturbances are thought to stem from fragmented sleep patterns associated with OSA, which adversely affect the brain’s capacity for emotional control and behavior regulation [6].

In addition to ED, pediatric OSA is frequently linked with anxiety and depression [7], compounding the challenges that children face in daily functioning and overall quality of life [8]. Timely diagnosis and intervention are crucial for mitigating these effects. Recent research has increasingly focused on the gut microbiota’s role in OSA, exploring the intricate interactions between gut health and neurobehavioral outcomes [9]. The microbiota–gut–brain axis offers a promising framework for understanding how gut microbiota influence neurodevelopment and emotional regulation through the modulation of neurotransmitters and systemic inflammatory responses [10,11].

Evidence suggests that children with OSA may experience alterations in their gut microbiome that could exacerbate or mitigate the metabolic, inflammatory, and autonomic disturbances commonly associated with the disorder [12,13,14]. These findings open new avenues for therapeutic interventions that target the gut microbiota, alongside traditional treatments like adenotonsillectomy and educational counseling—both of which have been shown to alleviate OSA symptoms and related behavioral challenges [15,16]. Our prior research has revealed significant changes in the gut microbiome following adenotonsillectomy, suggesting that microbiome-focused interventions could play a key role in improving treatment outcomes [14].

Considering the dynamic role of the gut microbiome during childhood development, interventions targeting microbiome modulation could hold promises for improving emotional and psychological health in children with OSA. Such strategies might involve dietary modifications, probiotics, or other lifestyle adjustments aimed at fostering a healthier gut microbiome, potentially reducing the risk or severity of ED [17]. Given the rising prevalence and long-term consequences of pediatric OSA, it is critical to examine its etiological factors and develop comprehensive treatment strategies.

Despite the growing recognition of the gut microbiota’s role in emotional and behavioral regulation, there remains limited understanding of how OSA affects gut microbiota composition and its potential role in ED. Additionally, there is a lack of integrated studies examining the interplay between gut microbiota, dietary habits, and autonomic function in pediatric OSA patients. Evidence is also scarce on the impact of adenotonsillectomy and educational interventions on microbiota-mediated emotional and behavioral outcomes in children with OSA.

This study aims to elucidate how OSA influences ED in children, focusing on the impact of adenotonsillectomy on the gut microbiota, dietary patterns, and sleep autonomic function. We hypothesize that (1) variations in gut microbiota composition, particularly in alpha and beta diversity, are associated with ED severity in children with OSA; and (2) adenotonsillectomy, combined with educational counseling, will significantly alter gut microbiota composition and diversity, with these changes correlating with improvements in ED scores. By adopting this comprehensive approach, the study seeks to enhance the understanding of the microbiota–gut–brain axis and to integrate microbiome-based interventions as adjuncts to traditional OSA treatments, ultimately improving emotional and behavioral outcomes for pediatric patients.

## 2. Materials and Methods

### 2.1. Study Population

This study involved a secondary data analysis of a prospective case-control study, including 66 pediatric patients with OSA who were treated at the Department of Otolaryngology, Head and Neck Surgery, Linkou Medical Center, Chang Gung Memorial Hospital, Taoyuan City, Taiwan, from March 2017 to January 2019 [18]. The study received approval from the Institutional Review Board of the Chang Gung Medical Foundation (No.: 201507279A3) and was conducted in accordance with the revised Declaration of Helsinki and STROBE guidelines [19,20]. Informed consent was obtained from the parents, and, where appropriate, directly from participants aged 6 and older [18].

Children aged 5–12 years diagnosed with OSA were considered eligible if they exhibited an apnea–hypopnea index (AHI) of ≥5.0 events per hour, or ≥2.0 events per hour when associated with morbidities such as elevated blood pressure or learning difficulties [18,21]. Exclusions were applied for children presenting with craniofacial abnormalities, neuromuscular disorders, chronic inflammatory conditions, recent infections, or acute gastroenteritis [18]. Additionally, for participants who had recently received antibiotic treatment due to infections or acute gastroenteritis, stool and blood samples were collected at least two weeks after the resolution of the acute episode to minimize the impact of antibiotics on microbiome analysis and inflammatory marker assessments [14,18,22].

The treatment involved adenotonsillectomy, followed by three educational sessions focusing on sleep hygiene, healthy eating, and exercise, conducted within three months post-surgery [16]. During these sessions, children and their caregivers received structured verbal recommendations on the following topics: (1) Sleep hygiene: Encouragement of adequate and early sleep, avoidance of caffeine after lunchtime, and refraining from large meals or vigorous physical activity before bedtime; (2) Healthy eating: Limiting sweetened beverages and fast food, and using the Daily Dietary Guidelines of Taiwan for Children as a reference [23]; (3) Regular exercise: Promoting outdoor and after-school physical activities and incorporating exercise training into daily routines; and (4) Nasal care: Recommending regular nasal saline irrigation [24]. Each session lasted 10–15 min and was designed to provide comprehensive care and support to the patients and their families. The adenotonsillectomy procedure details, along with the five-day post-treatment antibiotic regimen, are documented in a separate publication [25].

**Figure 1:**
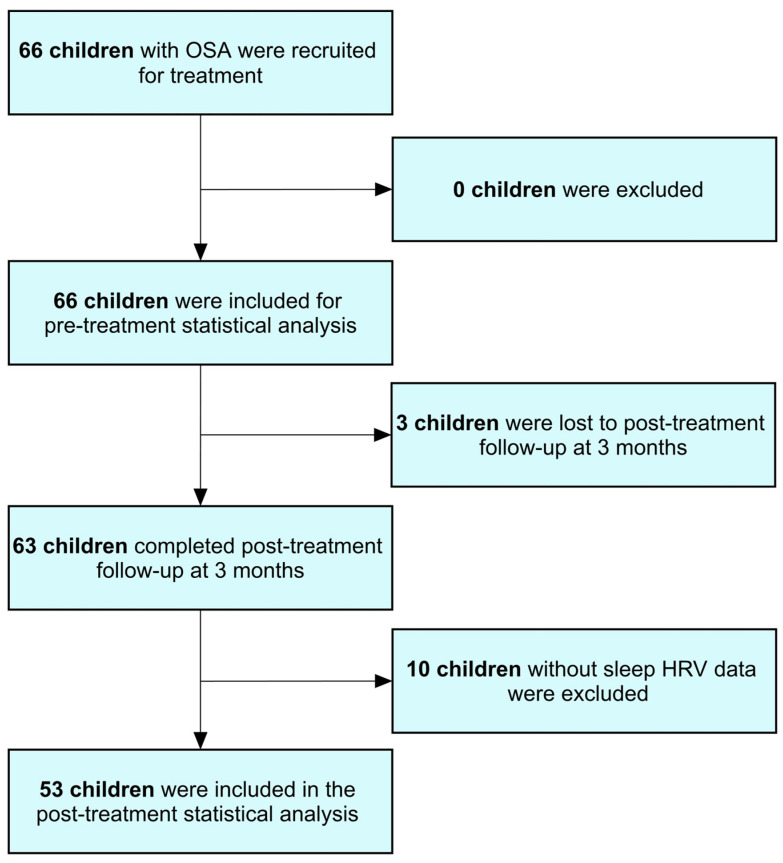
Case flow diagram. Abbreviations: HRV, heart rate variability; OSA, obstructive sleep apnea.

To assess the treatment’s impact, various tools were employed both before and three months after surgery. These included polysomnography for sleep monitoring, the OSA-18 quality of life questionnaire to assess patients’ ED, gut microbiome analysis to explore bacterial population changes, dietary assessments using the Short Food Frequency Questionnaire (SFFQ), and sleep heart rate variability (HRV) metrics to evaluate autonomic function. Additionally, research staff responsible for sample collection and study examinations did not participate in statistical analysis, ensuring unbiased interpretation of the data. Figure 1 illustrates the case flow diagram.

### 2.2. Endpoint Analysis

Primary outcomes included the ED score derived from the OSA-18 questionnaire and gut microbiota diversity measured through alpha and beta diversity indices. Alpha diversity was assessed using metrics such as observed species, Chao 1 index, and ACE index to gauge species richness, and indices such as Shannon, Simpson, InvSimpson, Fisher, Pielou, and coverage to evaluate overall community diversity within individual samples [26]. Beta diversity, evaluated using Bray–Curtis dissimilarity, examined variations in microbial community composition across samples [27].

Secondary outcomes included anthropometric variables, polysomnographic parameters, gut microbiome taxonomy, food frequency data, and sleep HRV metrics.

### 2.3. Polysomnography

Multiple indicators of OSA severity were assessed, including total sleep time, sleep stages, AHI, apnea index (AI), respiratory distress index (RDI), arousal index (ArI), mean peripheral oxygen saturation (SpO_2_), and minimum SpO_2_. In-lab polysomnography procedures were conducted following the 2012 American Academy of Sleep Medicine Manual guidelines [28]. Specific details of the polysomnography procedures used in this study have been published previously [18,29,30].

### 2.4. OSA-18 Questionnaire

Caregivers evaluated their children’s OSA-related quality of life using the Chinese version of the OSA-18 questionnaire [31]. The 18-item questionnaire, rated on a 7-point ordinal scale (1, none; 2, all the time) with a total score range of 18 to 126, has demonstrated excellent test–retest reliability [32]. It covers five key quality-of-life domains: sleep distress, physical symptoms, ED, daytime problems, and caregiver concerns. The ED domain, with a score range from 3 to 21, includes three specific behaviors: mood swings or temper tantrums, aggressive or hyperactive behavior, and discipline problems [32].

### 2.5. Gut Microbiome

Parents collected stool samples from participants in the morning, which were immediately snap-frozen and stored at −80 °C for later analysis [14,33]. Genomic DNA was extracted using a fecal DNA isolation kit (Cat# 12830-50, Mo Bio Laboratories, Carlsbad, CA, USA), and DNA quantity and quality were assessed using a NanoPhotometer P360 (Implen GmbH, München, Germany).

The V3–V4 regions of the bacterial 16 s rRNA gene were amplified using primers 341F and 806R, targeting two hypervariable regions to provide greater taxonomic resolution compared to single-region amplification (V3 or V4 alone) [34]. The PCR was conducted following Illumina’s 16 s Metagenomic Sequencing Library Preparation guidelines (Illumina, Inc., San Diego, CA, USA), with 12.5 ng of DNA, KAPA HiFi HotStart ReadyMix (Cat# 07958935001, Roche Diagnostics Corporation, Indianapolis, IN, USA), and a specific thermal cycling protocol: initial denaturation at 95°C for 3 min, followed by 25 cycles of 95 °C for 30 s, 55 °C for 30 s, and 72 °C for 30 s, with a final extension at 72 °C for 5 min.

Post-PCR, the products were confirmed via agarose gel electrophoresis, targeting a band around 500 base pairs. Products were purified using AMPure XP beads and subjected to a second PCR for adding Nextera XT Index Kit elements (Cat# FC-131-2001, Illumina, Inc.). Indexed products were validated with a Qubit 4.0 Fluorometer (Thermo Fisher Scientific Inc., Waltham, MA, USA) and the Qsep100 ™ system before pooling for sequencing on the Illumina MiSeq platform, producing paired 300-base-pair reads.

Post-sequencing, raw reads were demultiplexed using barcode information. The paired-end reads underwent primer and adapter sequence removal via the QIIME 2 cutadapt plugin (v2024.10) [35]. Amplicon sequence variant (ASV) [36] construction was performed using the QIIME 2 DADA2 plugin (v2024.10), which facilitated quality filtering, dereplication, and denoising while setting a maximum of two expected errors per read [37]. Trimming and filtering were set to a maximum of two expected errors per read. The DADA2 algorithm identified exact ASVs from the V3–V4 regions of the 16 s rRNA gene. Taxonomy classification for each ASV was conducted using the QIIME 2 feature-classifier plugin (v2024.10) [38] with the Silva database (release 138.2, 2024) as the reference [39]. To ensure accurate taxonomical annotation, the classifier was specifically trained with reference sequences limited to the V3–V4 regions targeted by the primer pair (341F/806R).

To assess sequence similarities among ASVs, a multiple sequence alignment was performed using QIIME 2′s MAFFT plugin [40]. Subsequently, a phylogenetic tree illustrating the relationships among representative ASV sequences was generated using QIIME 2′s FastTree plugin (v2.1) [41]. Diversity metrics, including alpha diversity and beta diversity, were calculated based on ASVs to ensure higher resolution and reproducibility of the results.

### 2.6. Short Food Frequency Questionnaire

Caregivers assessed children’s dietary habits over the past month using the SFFQ, documenting consumption frequencies for 23 food items, including a variety of beverages and foods such as different types of milk, orange juice, soft drinks, potatoes, vegetables, and typical snack foods [42]. Additionally, the questionnaire addressed the consumption of culturally specific items like rice and noodles [16].

Both participants and caregivers filled out the questionnaire, utilizing a frequency scale that varied by food type—from 0 (never) to 7 (four or more times per day) for solid foods, and 0 to 6 (seven glasses or more per day) for drinks. The questionnaire also included a simple yes/no question for the use of butter or margarine on bread. Validated for reliability, the SFFQ effectively ranks children’s food intake and dietary behaviors [16], ensuring accurate assessments of adherence to dietary guidelines [43], with reproducibility coefficients ranging from moderate to almost perfect (0.58–0.84) [44].

### 2.7. Sleep HRV Measurement

Electrocardiographic signals from polysomnography were analyzed using HRV software (ProfusionSLEEP™, version 4.5, Compumedics, Abbotsford, Australia). Experienced technicians manually reviewed these signals to correct any inaccuracies from automated annotations, such as loose leads, motion artifacts, and broken wires [29]. Consistent with established guidelines [45], frequency-domain indices were calculated, including total power, ultra-low frequency (ULF) (≤0.003 Hz), very low frequency (VLF) power (0.0033 to 0.04 Hz), low frequency (LF) power (0.04 to 0.15 Hz), high frequency (HF) power (0.15 to 0.4 Hz), and the LF/HF ratio, to assess various aspects of autonomic nervous system activity during sleep.

### 2.8. Sample Size Estimation

The pre-treatment sample size determination was based on prior research [46], focusing on the Shannon index as the primary outcome measure. Using G*Power software (version 3.1.9.2), the calculation considered an effect size of 0.92, a type I error rate (α) of 0.05, and a statistical power of 0.90 for a two-tailed test. The resulting minimum required sample size was 56 participants. To account for potential attrition and ensure robust statistical analysis, the target sample size was set at 66 participants.

### 2.9. Statistical Analysis

Statistical analyses were conducted using SPSS version 29.0 (IBM Corp., Armonk, NY, USA) and R software (v4.4.2; R Foundation for Statistical Computing, Vienna, Austria) [47]. The significance threshold was set at a two-tailed *p* < 0.05. The Shapiro–Wilk test was used to assess normality for major variables, and non-normal data were normalized as necessary.

Descriptive statistics included means, standard deviations, and medians for continuous variables, and frequencies for categorical variables. Between-group differences for continuous variables were evaluated using independent-samples *t*-tests, with Levene’s test confirming variance equality. Paired-samples *t*-tests were used for within-group comparisons. Categorical data comparisons were performed using Fisher’s exact test and McNemar’s test as appropriate. Correlations between study variables were analyzed using Pearson’s and point-biserial correlation tests. The Benjamini–Hochberg procedure was applied for multiple comparisons to control the false discovery rate and reduce the risk of Type I errors.

Multivariable linear regression models were developed to identify independent predictors from significant variables, employing backward selection with an inclusion threshold of *F* < 0.05. Variance inflation factors were maintained below 5 to minimize multicollinearity.

For microbiota data analysis, we utilized R software with the microeco package (v1.8.0) [48] for advanced ecological analysis and visualization. Microbial community analyses included the UpSet method for visualizing ASV intersections and permutational multivariate analysis of variance (PERMANOVA) with Benjamini–Hochberg correction to evaluate microbial diversity at the genus level [49].

Alpha diversity metrics assessed included observed species (species richness), ACE index (species richness), Shannon index (community diversity), Simpson index (community diversity), and Goods’ coverage (sequencing depth) [50]. Comparisons of alpha diversity metrics between groups were conducted using the Mann–Whitney *U* test. and were compared using the Mann–Whitney *U* test.

Beta diversity was evaluated using the Bray–Curtis dissimilarity index, which quantifies differences in microbial community composition across samples. Comparisons of beta diversity were performed using PERMANOVA [49].

Differential abundance analyses were performed using the multivariable association with linear models 2 (Maaslin2) package (v1.15.1) [51], incorporating adjustments for multiple comparisons through the Benjamini–Hochberg procedure to control the false discovery rate.

## 3. Results

### 3.1. Patient Characteristics

Sixty-six consecutive patients (boys 76%, *n* = 50; girls 24%, *n* = 16) with polysomnography-confirmed OSA were enrolled (Table 1). The mean age was 7.3 ± 2.2 years, the BMI was 19.01 ± 5.46 kg/m^2^, and the AHI was 14.70 ± 16.59 events/hour.

### 3.2. Differences in Patient Characteristics Between High and Low ED Groups

The mean score of ED score was 10.7 ± 4.0 and the mean total OSA-18 score was 75.7 ± 15.8. The study population was categorized into low ED (ED score < 11, *n* = 32) and high ED (ED score ≥ 11, *n* = 34) groups. The high ED group had a lower BMI and a higher total score (Table 1). There were no significant correlations between ED scores and patient characteristics or polysomnographic parameters. Additionally, total OSA-18 scores were significantly correlated with AHI (*r* = 0.27, *p* = 0.03), AI (*r* = 0.26, *p* = 0.04), and RDI (*r* = 0.27, *p* = 0.045). However, these correlations did not remain significant after applying the Benjamini–Hochberg correction.

### 3.3. Differences in Gut Microbiome Composition Between High and Low ED Groups

**Figure 2:**
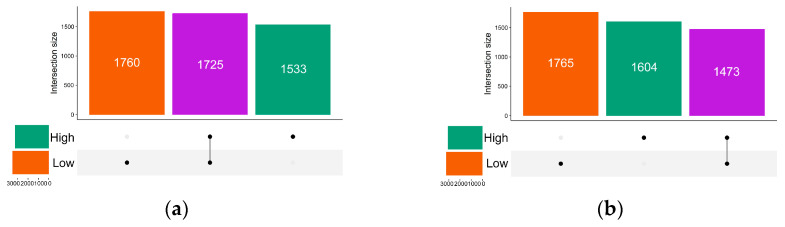
Distributions of the gut microbiota amplicon sequence variants (ASVs) between high emotional distress (ED) and low ED groups in pediatric obstructive sleep apnea. (**a**) The UpSet plot illustrates the unique and shared ASVs between the low ED group (3485 ASVs) and the high ED group (3258 ASVs) at the pre-treatment stage. (**b**) The UpSet plot illustrates the unique and shared ASVs between the low ED group (3238 ASVs) and the high ED group (3077 ASVs) at the post-treatment stage.

**Figure 3:**
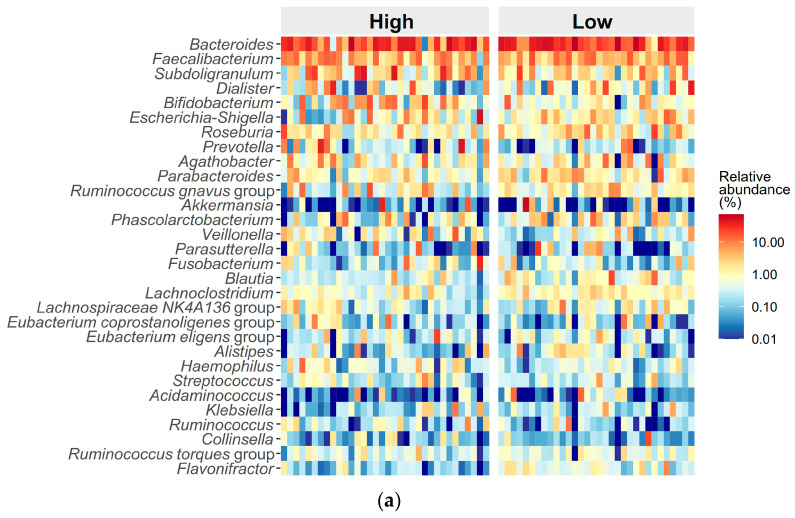

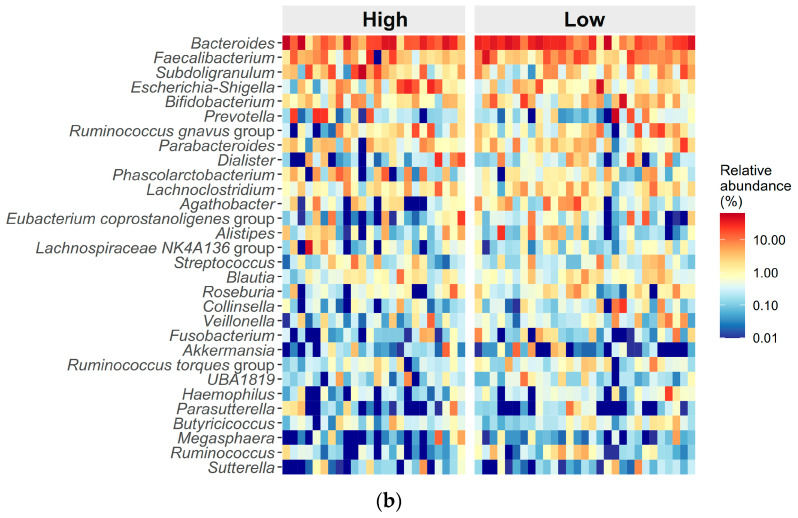
Gut microbial distributions of the top 30 genera between the high and low emotional distress (ED) groups in pediatric obstructive sleep apnea. (**a**) The heatmap illustrates significant differences in the microbial distribution of the top 30 genera between the high and low ED groups at the pre-treatment stage, with statistical significance confirmed by permutational multivariate analysis of variance with Benjamini–Hochberg correction (*p* = 0.01). (**b**) The heatmap illustrates a similar microbial distribution of the top 30 genera between the high and low ED groups at the post-treatment stage (*p* = 0.33).

The high ED group had 3258 identified ASVs while the low ED group had 3485 ASVs, sharing 1725 ASVs (Figure 2a). According to our previous study, V3–V4 region 16 s rRNA sequencing provided comparable taxonomic profile resolutions from the phylum to the genus levels as full-length 16 s rRNA sequencing [33]. Therefore, we further focused the microbiota analysis on the genus level. Using PERMANOVA, the distributions of the top 30 genera between the high ED group and the low ED group were significantly different (*p* = 0.01; Figure 3a).

**Figure 4:**
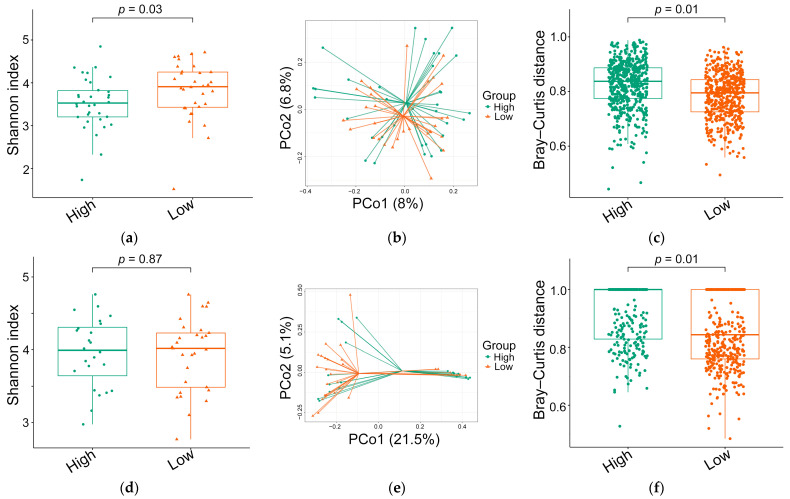
Gut microbiome stratification between high and low emotional distress (ED) groups in pediatric obstructive sleep apnea. (**a**) The scatter boxplot illustrates a significantly lower Shannon index in the high ED group compared to the low ED group at the pre-treatment stage (*p* = 0.03). (**b**) Principal coordinates analysis (PCoA) highlights significant differences in Bray–Curtis distances between the two ED groups at the pre-treatment stage. (**c**) The scatter boxplot shows significantly greater microbial community variability in the high ED group compared to the low ED group at the pre-treatment stage (*p* = 0.01). (**d**) The scatter boxplot indicates comparable Shannon indices between the two ED groups at the post-treatment stage (*p* = 0.87). (**e**) PCoA highlights differences in Bray–Curtis distances between the two ED groups at the post-treatment stage. (**f**) The scatter boxplot shows a significant increase in microbial community variability within the high ED group compared to the low ED group at the post-treatment stage (*p* = 0.01). Shannon indices were compared using the independent-samples *t*-test, and Bray–Curtis distances were compared using permutational multivariate analysis of variance with Benjamini–Hochberg correction.

Alpha diversity metrics indicated that the observed species and coverage were comparable between the high and low ED groups (Table 2). However, the Shannon index was significantly lower in the high ED group compared to the low ED group (Figure 4a), while other alpha diversity indices showed no significant differences. The relative abundances of *Blautia* (*p* = 0.01), *Lachnoclostridium* (*p* < 0.001), and *Flavonifractor* (*p* < 0.001) were significantly lower in the high ED group compared to the low ED group. After applying the Benjamini–Hochberg correction, the differences in *Lachnoclostridium* and *Flavonifractor* remained statistically significant.

Before applying the Benjamini–Hochberg correction, the Shannon and Simpson indices were positively associated with male sex (*r* = 0.26 and 0.27; *p* = 0.03 and 0.03, respectively), and the Simpson index was inversely associated with AHI (*r* = −0.27, *p* = 0.03). Additionally, the observed species and ACE index were inversely associated with AI (*r* = −0.27 and −0.26; *p* = 0.03 and 0.03, respectively), and the observed species and Shannon index were inversely associated with ED score (*r* = −0.25 and −0.31; *p* = 0.045 and 0.01, respectively). However, none of these relationships remained significant after applying the Benjamini–Hochberg correction.

The principal coordinates analysis and beta diversity assessment using the Bray–Curtis distance indicated significant differences in the gut microbiota between both ED groups (*p* = 0.01) (Figure 4b,c).

Using Maaslin2 analysis with the Benjamini–Hochberg correction, six marker microbes among the top 30 genera were significantly associated with different ED levels: *Flavonifractor* (coefficient = −0.04, *p* < 0.001), *Bifidobacterium* (coefficient = 0.08, *p* = 0.01), *Lachnoclostridium* (coefficient = −0.03, *p* = 0.01), *Parabacteroides* (coefficient = −0.04, *p* = 0.03), *Blautia* (coefficient = −0.04, *p* = 0.03), and *Haemophilus* (coefficient = 0.03, *p* = 0.046). Notably, the relative abundance of *Bifidobacterium* was higher in the high ED group compared to the low ED group, whereas the relative abundances of *Flavonifractor*, *Lachnoclostridium*, *Parabacteroides,* and *Blautia* were lower in the high ED group (Table 2).

**Figure 5:**
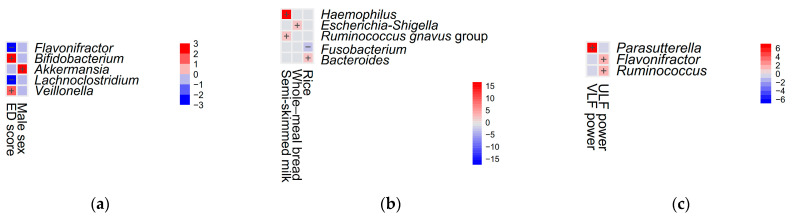
Heatmap of significant associations (−log(q value) × sign(coefficient)) between the top 30 genera and variables of interest. (**a**) Heatmap showing significant associations across five genera, emotional distress (ED) score, and male sex. (**b**) Heatmap depicting significant associations across five genera, semi-skimmed milk, whole-meal bread, and rice consumption. (**c**) Heatmap illustrating significant associations across three genera, very low frequency (VLF) power, and ultra-low frequency (ULF) power. Positive signs indicate enrichment, and negative signs indicate depletion of the corresponding genera relative to increasing variables of interest. The analysis was conducted using the Maaslin2 R package (v1.15.1).

Furthermore, Maaslin2 analysis revealed four genera associated with ED scores: *Bifidobacterium* (coefficient = 0.01, *p* = 0.01) and *Veillonella* (coefficient = 0.01, *p* = 0.02) showed positive associations, while *Flavonifractor* (coefficient = −0.005, *p* = 0.048) and *Lachnoclostridium* (coefficient = −0.004, *p* = 0.01) displayed negative associations. Additionally, a positive association between female sex and the genus *Akkermansia* (coefficient = −0.11, *p* = 0.01) was identified (Figure 5a).

### 3.4. Differences in Food Frequency Between High and Low ED Groups

The SFFQ identified notable dietary differences between children with high and low ED scores. Children with higher ED scores reported more frequent consumption of orange juice, boiled potatoes, pizza, and butter/margarine on bread (Table 3); however, these associations lost significance after applying the Benjamini–Hochberg correction.

The ED score was positively associated with consumption of savory snacks (*r* = 0.24, *p* = 0.049). The total OSA-18 score was significantly related to sweets (*r* = 0.42, *p* <0.001) and savory snacks (*r* = 0.42, *p* <0.001). After Benjamini–Hochberg correction, the associations between total OSA-18 scores and sweets, as well as savory snacks, remained significant.

Maaslin2 analysis revealed that semi-skimmed milk consumption was positively correlated with the relative abundance of gut bacteria, including *Haemophilus* (coefficient = 0.31, *p* < 0.001) and the *Ruminococcus gnavus* group (coefficient = 0.26, *p* = 0.004). Similarly, whole-meal bread consumption showed a positive association with the relative abundance of *Escherichia-Shigella* (coefficient = 0.04, *p* = 0.003). Rice consumption was positively associated with *Bacteroides* (coefficient = 0.08, *p* = 0.01) and inversely associated with *Fusobacterium* (coefficient = −0.03, *p* = 0.004) (Figure 5b).

### 3.5. Differences in Sleep Heart Rate Variability Metrics Between High and Low ED Groups

Our results indicate that the high ED group exhibited significantly lower values for total power and ULF power compared to the low ED group, as shown in Table 4. However, these differences lost significance after applying the Benjamini–Hochberg correction.

The ED score was initially found to be negatively associated with ULF power (*r* = −0.33, *p* = 0.01); however, this association also became non-significant following the Benjamini–Hochberg correction. In contrast, total OSA-18 scores did not show any significant relationships with HRV metrics.

Maaslin2 analysis further revealed positive correlations between ULF power and the relative abundance of specific gut bacteria, including *Flavonifractor* (coefficient = 0.00001, *p* = 0.01) and *Ruminococcus* (coefficient = 0.00001, *p* = 0.004). Additionally, VLF power demonstrated a positive association with the relative abundance of *Parasutterella* (coefficient = 0.00002, *p* < 0.001) (Figure 5c).

### 3.6. Multiple Linear Regression Models for ED Score Estimation

To develop predictive models for ED score estimation, the Shannon index and significant genera from the top 30 identified through Maaslin2 analysis, including *Veillonella*, *Bifidobacterium*, *Flavonifractor*, and *Lachnoclostridium*, were incorporated into multiple linear regression models using backward selection.

In Model 1, the Shannon index, along with the relative abundances of *Veillonella*, *Bifidobacterium*, and *Flavonifractor*, were retained as significant predictors. This model explained 28% of the variance in ED scores (Table 5).

Model 2 further expanded the analysis by integrating significant genera with additional relevant variables of interest. Independent predictors in this model included the relative abundances of *Bifidobacterium* and *Flavonifractor*, as well as ULF power. This model accounted for 29% of the variance in ED scores (Table 5).

### 3.7. Changes in Variables of Interest After Treatment

At the 3-month post-treatment follow-up, 63 participants (95%) completed clinical evaluations, and 53 participants (80%) underwent assessments of gut microbiome and sleep HRV metrics (Figure 1). Significant post-treatment improvements were observed, including increases in BMI, mean SpO_2_, and minimum SpO_2_, alongside reductions in AHI, AI, RDI, ArI, and both ED and total scores of the OSA-18 questionnaire, with or without adjustment (Table 1).

Gut microbiome analysis showed significant increases in the Shannon index, Simpson index, and coverage of alpha diversity metrics, along with an increase in the Bray–Curtis distance of beta diversity. In contrast, observed species and the ACE index significantly decreased (Table 2). While the relative abundance of *Lachnoclostridium* significantly increased and *Bacteroides* and *Roseburia* significantly decreased before correction, none of these differences remained significant after the Benjamini–Hochberg correction.

Regarding dietary habits, the frequencies of full-fat milk drinking and potato chip consumption significantly increased post-treatment; however, these changes also lost significance after the Benjamini–Hochberg correction (Table 3). Sleep HRV metrics, including total power, ULF power, LF power, and HF power, significantly decreased. After correction, reductions in total power, LF power, and HF power remained significant (Table 4).

### 3.8. Comparing Post-Treatment Variables of Interest Between Pre-Treatment ED Groups

The post-treatment ED score in the pre-treatment high ED group remained significantly higher than that in the low ED group. Similarly, the total OSA-18 questionnaire score in the pre-treatment high ED group was significantly higher compared to the low ED group, and these differences remained statistically significant after correction (Table 1).

The number of identified ASVs decreased in both groups post-treatment, from 3258 to 3077 in the high ED group and from 3485 to 3238 in the low ED group. Both groups shared 1473 ASVs (Figure 3b). The distribution of the top 30 genera between the high and low ED groups was comparable (*p* = 0.33; Figure 4b). Both groups demonstrated similar Shannon indexes (Figure 4d), Simpson indexes, and coverage values. However, the high ED group had significantly lower observed species and ACE index values compared to the low ED group. The Bray–Curtis distance remained significantly higher in the high ED group compared to the low ED group (Figure 4e,f). While the relative abundances of *Haemophilus* and *Ruminococcus* were significantly lower in the high ED group, these differences did not persist after correction (Table 2).

Using Maaslin2 analysis with the Benjamini–Hochberg correction, only one marker microbe, *Ruminococcus* (coefficient = −0.03, *p* = 0.03), was identified as being significantly associated with high ED levels. Additionally, the relative abundance of *Subdoligranulum* (coefficient = 0.01, *p* = 0.01) was significantly associated with post-treatment ED scores.

Before correction, the frequency of fish consumption for dinner was significantly higher in the high ED group (Table 3), and total power and VLF power in the high ED group were significantly lower than those in the low ED group (Table 4). However, these differences were no longer statistically significant after correction.

### 3.9. Multiple Linear Regression Models for Change in ED Score

The change in ED score was significantly associated with several factors. Positive correlations were observed with changes in the relative abundances of *Agathobacter* (*r* = 0.30, *p* = 0.03) and *Veillonella* (*r* = 0.30, p = 0.03), as well as changes in the consumption of low-fat milk (*r* = 0.32, *p* = 0.02) and skimmed milk (*r* = 0.30, *p* = 0.03). Conversely, negative correlations were noted between the change in ED score and changes in the relative abundance of *Flavonifractor* (*r* = −0.28, *p* = 0.04) and LF power (*r* = −0.33, *p* = 0.03).

Using multiple linear regression models with backward selection, two multifactorial models were developed to predict changes in ED scores (Table 5):

Model 3 focused on microbial variables, identifying changes in the relative abundances of *Veillonella* and *Agathobacter* as significant predictors. This model explained 14% of the variance in ED score changes.

Model 4 incorporated microbial variables and physiological markers, with significant predictors including changes in the relative abundances of *Veillonella* and LF power. This model explained 20% of the variance in ED score changes.

## 4. Discussion

Our study underscores the multifaceted interplay between OSA, ED, gut microbiota composition, dietary habits, and physiological factors in pediatric patients. Although AHI and other polysomnographic parameters were significantly correlated with total OSA-18 scores, these parameters did not significantly correlate with ED component scores, suggesting different underlying mechanisms. Furthermore, reductions in ED scores following adenotonsillectomy and educational counseling did not correspond with changes in polysomnographic parameters. This result aligns with prior research showing that changes in AHI do not correlate with improvements in attention-deficit/hyperactivity disorder (ADHD) scores [15]. It suggests that polysomnographic metrics, while valuable for assessing sleep-related disturbances, may not adequately capture the emotional and behavioral dimensions represented by the ED component of the OSA-18 questionnaire.

Our analysis identified significant differences in gut microbiota diversity between the high and low ED groups. The high ED group exhibited lower pre-treatment Shannon index values for alpha diversity, reflecting reduced overall community diversity. This finding is consistent with previous studies that reported diminished gut microbiota alpha diversity in children with ADHD [46,52]. Furthermore, the significant difference in the pre-treatment Bray–Curtis distance for beta diversity between the two ED groups aligns with findings from similar research in pediatric ADHD [53]. Notably, alpha diversity was not correlated with polysomnographic parameters, suggesting its influence on ED severity may occur through mechanisms independent of direct OSA effects. However, multivariable linear regression modeling revealed that several factors contributed significantly to pre-treatment ED scores. In Model 1, the Shannon index, along with the genera *Veillonella*, *Bifidobacterium*, and *Flavonifractor*, emerged as key predictors. In Model 2, the relative abundances of *Bifidobacterium* and *Flavonifractor*, along with ULF power, were identified as significant contributors to ED scores.

After OSA treatment, differences in alpha diversity between high and low ED groups diminished, becoming statistically insignificant. However, the difference in post-treatment Bray–Curtis distance remained significant. Additionally, while ED scores in the high ED group decreased significantly post-treatment, they remained higher than those in the low ED group. These findings suggest that alpha diversity may be a responsive component of ED to OSA treatment, while beta diversity may represent a non-responsive component.

Multivariable linear regression models identified changes in the relative abundances of *Veillonella* and *Agathobacter* in Model 3, and changes in the relative abundance of *Veillonella* and LF power in Model 4, as significant predictors of post-treatment changes in ED scores, highlighting their potential roles in mediating emotional outcomes following OSA intervention.

Taken together, we further focused on these specific genera (*Veillonella, Bifidobacterium*, *Flavonifractor*, and *Agathobacter*) and autonomic nervous system function markers (ULF power and LF power) to explore their potential mechanisms in influencing ED, as highlighted in the current literature.

The *Veillonella* genus, a strictly anaerobic Gram-negative cocci that metabolizes lactate into weaker acids like acetate and propionate, is prevalent in the human intestines and oral mucosa [54]. Research suggests that increased levels of *Veillonella* and its metabolic products can exacerbate negative emotions in individuals with irritable bowel syndrome [55] and have been linked to non-social fear behaviors in infants [56]. Propionic acid, a metabolic byproduct of *Veillonella*, has been implicated in mediating social deficits and anxiety-like behaviors in animal studies [57]. Moreover, prebiotics and probiotics that enhance propionic acid levels have demonstrated benefits in reducing emotional dysregulation in patients with ADHD [58]. These findings suggest that modulating *Veillonella* levels could influence ED in children with OSA, presenting an opportunity for further investigation into its potential as a therapeutic target.

*Bifidobacterium*, an anaerobic Gram-positive bacterium involved in oligosaccharide metabolism and the production of acetate and lactate, is prevalent in the human intestine [59]. Variations in gut *Bifidobacterium* profiles are frequently observed in children and adolescents with psychiatric disorders [60]. For instance, an increased abundance of *Bifidobacterium*, associated with enhanced biosynthesis of the dopamine precursor phenylalanine, has been reported in the gut microbiome of children with ADHD [61]. Conversely, children with autism spectrum disorder (ASD) exhibit significantly lower levels of *Bifidobacterium* compared to healthy children [17]. In our study cohort, although the relative abundance of *Bifidobacterium* was slightly higher in the high ED group, its elevated levels emerged as a significant predictor of increased ED scores.

Supplementation with *Bifidobacterium bifidum* (Bf-688) has been shown to improve neuropsychological performance in children with ADHD, potentially by altering gut microbiota composition and reducing N-Glycan biosynthesis [62]. Similarly, interventions using probiotic *Bifidobacterium* and fructo-oligosaccharides have demonstrated the ability to modulate gut microbiota, increase short-chain fatty acid levels, and enhance serotonin production, resulting in improvements in ASD symptoms [63]. These findings suggest that modulating gut *Bifidobacterium* levels may play a crucial role in addressing emotional and behavioral issues in children with OSA.

*Flavonifractor*, a strictly anaerobic rod-shaped bacterium within the phylum *Firmicutes*, is known for its role in flavonoid degradation and harbors variants with diverse metabolic and morphological traits, often exhibiting negative co-occurrence patterns [64]. The abundance of *Flavonifractor* has been linked to negative emotional valence in the human functional domain [65]. Similar to its reduced levels observed in children with ASD and chronic gastrointestinal symptoms [66], our study found a lower relative abundance of *Flavonifractor* in children with OSA and high ED traits compared to those with low ED traits. While direct associations are lacking, evidence from animal models of acute liver injury suggests that supplemental *Bifidobacterium* can reduce gut flora dysbiosis, including modifications to *Flavonifractor* levels, and attenuate systemic inflammation [67]. This finding indicates the potential of *Bifidobacterium* supplementation to influence gut *Flavonifractor* composition and systemic health, which could be further explored in the pediatric OSA population.

*Agathobacter*, a member of the *Lachnospiraceae* family, is known for its ability to produce butyrate in the gastrointestinal tract [68]. This genus has been implicated in various mood disorders, with elevated levels linked to symptoms of withdrawal and depression in children with ADHD [69]. It has also been proposed as a potential biomarker for predicting antidepressant efficacy in the elderly [70]. Conversely, some studies suggest that reduced levels of *Agathobacter* correlate with depressive symptoms in women [71] and that increasing its abundance through specific probiotics can alleviate anxiety and depression in patients with irritable bowel syndrome [72], underscoring its complex role in mood regulation [73].

The production of butyric acid by *Agathobacter* may offer protective effects against depressive symptoms by reducing intestinal permeability associated with depression and modulating the hypothalamic–pituitary–adrenal axis [74,75]. In our cohort, increased *Agathobacter* levels were associated with heightened ED scores post-treatment, particularly regarding mood swings, temper tantrums, and aggressive or hyperactive behavior. This finding suggests a multifaceted role for *Agathobacter* in modulating emotional responses in pediatric OSA. Further research is needed to elucidate whether *Agathobacter*’s influence on emotional health is protective or exacerbatory and to explore its therapeutic potential in this context.

Furthermore, our analysis indicates that both ULF and LF powers may influence ED scores. Specifically, an increase in ULF (≤0.003 Hz) power was associated with decreased ED scores prior to OSA treatment. ULF power, which is linked to very low frequency biological processes such as circadian rhythms, neuroendocrine activity, and thermoregulation [76,77], is less understood than HF and LF powers but has been recognized as a significant predictor of risk in both cardiovascular and psychiatric disorders [78,79]. Circadian rhythms, in particular, are thought to be a primary driver of ULF power in studies concerning psychiatric disorders [79,80], suggesting a critical role for ULF power in autonomic nervous system regulation [81,82].

Previous studies have noted disruptions in sympathetic activity related to emotional reactivity and parasympathetic activity in emotion regulation among children with ADHD [83]. Our findings imply that children with OSA may display adaptive parasympathetic-based regulation, potentially mitigating the effects of heightened sympathetic-linked disruptions. This adaptive regulation could help explain why higher ULF power correlates with lower ED scores, underscoring the potential benefits of targeting autonomic function as part of a comprehensive approach to managing emotional disturbances in pediatric OSA.

The LF (range, 0.04–0.15 Hz) component of the HRV spectrum is influenced by both the parasympathetic and sympathetic nervous systems [45,84]. Although the sympathetic nervous system generally does not produce rhythms much above 0.1 Hz, the parasympathetic system can influence heart rhythms down to 0.05 Hz [85]. In our previous research, we observed significantly higher sleep SDNN and total power (total capacity of the regulation system), and VLF power (sympathetic activity) in children with OSA compared to normal values [29]. In this study, we found that changes in sleep LF power were inversely correlated with changes in ED scores post-OSA-treatment, although the difference in LF power between the high and low ED groups was not significant.

Changes in LF power can reflect the body’s response to stress and emotional challenges. For example, an increase in sympathetic nervous system activity, which may accompany emotional dysregulation, can lead to changes in LF power [86]. However, a meta-analysis suggested that the most frequently reported factor associated with stress is low parasympathetic activity, characterized by a decrease in HF power and an increase in the LF band [87]. Thus, ED in children with OSA can influence both sympathetic and parasympathetic activities, which are not solely explained by heightened sympathetic activation.

Sleep is often seen as a period for processing and resolving distressing experiences. Low levels of acetylcholine during non-rapid eye movement and reduced levels of noradrenaline during rapid eye movement sleep provide a unique window for the plasticity of neuronal representations of emotional memories, potentially resolving associated ED [88]. Effective OSA management, which reduces disease severity, may increase acetylcholine-associated parasympathetic activity and decrease noradrenaline levels during sleep [89]. These changes might enhance overnight neuronal plasticity and adaptability to emotional dysregulation, potentially explaining the inverse relationship between changes in LF power and ED scores.

### Strengths and Limitations

This study underscores the complex interplay between sleep disorders like OSA, gut microbiota composition alterations, dietary factors, systemic inflammation, and autonomic function in influencing emotional and behavioral outcomes in children. While the findings suggest that targeting these interconnected factors could provide therapeutic benefits, several limitations should be acknowledged.

First, the small sample size limits the generalizability of the results. A larger, more diverse cohort would be essential for confirming these associations. Additionally, the observational nature of the study prevents us from drawing definitive conclusions about causality. It remains unclear whether changes in the gut microbiota directly influence ED or whether ED, along with its associated behavioral and physiological changes, alters the gut microbiota composition. The bidirectional relationship between the gut microbiota and the brain, mediated through the microbiota–gut–brain axis, further complicates causal interpretations [90]. Randomized controlled trials and mechanistic studies are needed to delineate whether these microbiota changes are a cause, a consequence, or a cofactor in ED development in pediatric OSA.

Second, the reliance on caregiver-reported tools like the OSA-18 questionnaire and SFFQ introduces biases that may affect the accuracy of findings. The OSA-18, while widely used, may over- or underestimate symptoms due to caregiver perceptions and lacks sensitivity for detecting nuanced ED symptoms like hyperactivity and mood regulation [91]. Similarly, the SFFQ is prone to recall bias due to caregivers’ memory constraints or social desirability bias [92], and does not account for dietary complexities, such as preparation methods and portion sizes, which influence gut microbiota composition. Future studies should use more objective tools, such as structured behavioral assessments, clinician-administered evaluations, direct dietary observations, or biomarkers, to improve data reliability and better elucidate the links between diet, gut microbiota, and ED in children with OSA.

Third, the high-throughput sequencing of the V3–V4 regions of the 16 s rRNA gene used in this study has inherent limitations in taxonomic resolution compared to full-length 16 s rRNA gene sequencing [33,93]. Although the V3–V4 regions offer a practical balance of cost, data yield, and classification accuracy [33], future studies should consider employing full-length 16 s rRNA gene sequencing or other advanced methods to enable more precise species-level analyses and improve the depth of microbiome characterization.

Fourth, although the counseling sessions were standardized in terms of content and duration (10–15 min per session) and delivered by trained staff, we did not objectively measure participants’ engagement or adherence to the recommended practices for sleep hygiene, diet, and exercise. This lack of monitoring introduces variability that could have influenced the effectiveness of the counseling and its contribution to the observed outcomes. Future studies should incorporate strategies to assess and enhance consistency in delivery and adherence, such as adherence-monitoring tools, structured follow-up assessments, or digital tracking systems [94]. Additionally, evaluating the impact of individual engagement levels on intervention outcomes could provide valuable insights for optimizing and tailoring these interventions.

Finally, while this study opens the door to exploring microbiome-targeting strategies as potential treatments or adjunct therapies for OSA, further research is needed to establish clear mechanistic links. Future studies should explore how changes in gut microbiota composition influence autonomic function and directly modulate ED in children with OSA. The observed associations between gut microbiota changes and ED improvements are preliminary, underscoring the need for longitudinal studies with larger, more diverse cohorts to confirm causative relationships between microbiota alterations and emotional and behavioral outcomes. Additionally, randomized controlled trials should evaluate the efficacy of microbiota-targeted interventions, such as dietary modifications, prebiotics, probiotics, and synbiotics, in reducing ED in children with OSA.

Moreover, advanced omics approaches such as metagenomics, transcriptomics, and metabolomics could uncover specific microbial pathways and metabolites contributing to these effects [95]. The integration of multi-omics data with detailed assessments of autonomic nervous system function and systemic inflammation could provide a more holistic understanding of the microbiota–gut–brain axis in pediatric OSA. Exploring interactions between microbiota modulation and existing OSA treatments, such as adenotonsillectomy and behavioral therapies, could further refine therapeutic strategies. These efforts will enable the development of personalized, microbiome-focused therapies to improve outcomes for children with OSA.

## 5. Conclusions

This study highlights the significant role of specific gut bacteria, including alpha and beta diversity, *Veillonella*, *Bifidobacterium*, and *Flavonifractor*, in the ED observed in children with OSA. Post-treatment changes in gut microbiota composition, particularly increases in *Veillonella* and *Agathobacter*, were closely associated with improvements in ED scores. These findings underscore the potential of microbiota-targeted strategies, such as dietary modifications and probiotic or prebiotic supplementation, as complementary approaches to traditional OSA treatments. Future research should prioritize investigating specific probiotic strains that modulate *Veillonella* and *Agathobacter* and testing dietary interventions aimed at altering gut microbiota composition. Furthermore, HRV-guided interventions may offer additional benefits for mood and behavioral regulation in this population. Randomized controlled trials are needed to assess the efficacy of these approaches and clarify the mechanistic pathways underlying the microbiota–gut–brain axis. This research lays a foundation for innovative therapies targeting the gut microbiome to enhance emotional and behavioral outcomes in pediatric OSA patients.

## Figures and Tables

**Table 1:** Demographic characteristics, polysomnographic parameters, and key OSA-18 questionnaire scores before and after treatment for pediatric obstructive sleep apnea.

Variable	Pre-Treatment Status (*n* = 66)	Emotional Distress		Post-Treatment Status (*n* = 53)	Emotional Distress		*p*-Value ^1^	*p*-Value ^2^	*p*-Value ^3^
		Low (*n* = 32)	High (*n* = 34)		Low (*n* = 29)	High (*n* = 24)			
Demographic characteristics									
Age at enrollment, years, mean (median)	7.3 ± 2.2 (7)	7.3 ± 2.3 (7)	7.3 ± 2.1 (6)	7.5 ± 2.3 (7)	7.5 ± 2.4 (7)	7.6 ± 2.2 (7)	0.88	0.78	
Gender (boy), % (*n*)	76 (50)	84 (27)	23 (68)	77 (41)	83 (24)	71 (17)	0.15	0.34	
BMI, kg/m^2^, mean (median)	19.01 ± 5.46 (17.4)	20.36 ± 5.91 (19.0)	17.74 ± 4.73 (15.7)	20.33 ± 6.12 (18.7)	21.90 ± 6.49 (19.3)	19.28 ± 5.61 (16.8)	0.049	0.26	<0.001
Polysomnographic parameters									
AHI, events/hour, mean (median)	14.70 ± 16.59 (8.5)	18.20 ± 19.54 (9.6)	11.41 ± 12.67 (5.8)	3.38 ± 3.33 (2.4)	3.52 ± 3.44 (2.5)	3.21 ± 3.27 (2.3)	0.17	0.74	<0.001 *
AI, events/hour, mean (median)	4.63 ± 8.37 (2.0)	5.58 ± 10.41 (1.9)	3.74 ± 5.86 (2.2)	1.27 ± 1.53 (0.8)	1.33 ± 1.38 (0.9)	1.20 ± 1.71 (0.8)	0.30	0.77	0.002 *
RDI, events/hour, mean (median)	15.72 ± 17.05 (9.6)	18.93 ± 20.03 (10.6)	12.84 ± 13.54 (9.5)	3.93 ± 3.36 (3.2)	4.30 ± 3.51 (3.9)	3.49 ± 3.19 (2.4)	0.18	0.39	<0.001 *
ArI, events/hour, mean (median)	14.75 ± 11.77 (10.4)	17.55 ± 14.33 (14.1)	12.13 ± 8.13 (9.0)	8.35 ± 3.80 (7.5)	8.77 ± 3.14 (8.2)	7.85 ± 4.48 (6.6)	0.06	0.38	<0.00 *
Mean SpO_2_, %, mean (median)	97.2 ± 1.4 (97)	96.9 ± 1.7 (97)	97.5 ± 0.9 (98)	97.7 ± 0.8 (98)	97.7 ± 0.7 (98)	97.7 ± 1.0 (98)	0.28	0.75	0.0 *
Minimum SpO_2_, %, mean (median)	87.8 ± 7.2 (90)	86.5 ± 8.4 (89)	89.1 ± 5.7 (90)	91.2 ± 3.7 (92)	90.3 ± 4.3 (91)	92.2 ± 2.5 (93)	0.16	0.06	<0.001 *
OSA-18 questionnaire scores									
ED, score, mean (median)	10.7 ± 4.0 (11)	7.3 ± 2.0 (7)	13.9 ± 2.5 (14)	8.4 ± 3.9 (8)	6.8 ± 3.3 (6)	10.3 ± 3.7 (10)	<0.001 *	<0.001 *	<0.001 *
Total, score, mean (median)	75.7 ± 15.8 (77)	68.7 ± 13.8 (67)	82.4 ± 14.8 (85)	45.2 ± 1.6 (46)	41.2 ± 10.6 (42)	49.9 ± 13.3 (51)	<0.001 *	0.01 *	<0.001 *

Data are presented as mean ± standard deviation (median) or percentage (*n*) as appropriate. Abbreviations: AHI, apnea–hypopnea index; AI, apnea index; ArI, arousal index; BMI, body mass index; ED, emotional distress; OSA, obstructive sleep apnea; RDI, respiratory distress index; SpO2, peripheral oxygen saturation. ^1^ *p*-Values were calculated using independent-samples *t*-test for continuous variables of demographic variables, one-way multivariate analysis of variance for continuous variables of polysomnographic parameters and OSA-18 domain and total scores, and Fisher's exact test for categorical variables between pre-treatment groups. ^2^ *p*-Values were calculated using the same statistical methods between post-treatment groups. ^3^ *p*-Values were calculated using paired-samples *t*-test for continuous variables. * *p*-Values were found to be significant after adjusting for multiple comparisons using the Benjamini–Hochberg procedure.

**Table 2:** Gut microbiome before and after treatment for pediatric obstructive sleep apnea.

Variable	Pre-Treatment Status (*n* = 66)	Emotional Distress		Post-Treatment Status (*n* = 53)	Emotional Distress		*p*-Value ^1^	*p*-Value ^2^	*p*-Value ^3^
		Low (*n* = 32)	High (*n* = 34)		Low (*n* = 29)	High (*n* = 24)			
Alpha diversity metrics									
Observed species, mean (median)	321.8 ± 117.5 (319)	347.2 ± 116.6 (384)	297.8 ± 115.0 (274)	278.8 ± 75.9 (268)	293.1 ± 65.6 (292)	261.5 ± 84.8 (246)	0.09	0.04	0.01
ACE index, mean (median)	375.7 ± 146.6 (362)	402.1 ± 141.4 (439)	350.8 ± 179.0 (306)	306.8 ± 104.1 (280)	321.3 ± 79.5 (313)	289.2 ± 127.8 (263)	0.16	0.03	0.003
Shannon index, mean (median)	3.66 ± 0.65 (3.7)	3.82 ± 0.67 (3.9)	3.52 ± 0.61 (3.5)	3.94 ± 0.48 (4.0)	3.93 ± 0.50 (4.0)	3.96 ± 0.48 (4.0)	0.03	0.87	0.035
Simpson index, mean (median)	0.91 ± 0.08 (0.9)	0.92 ± 0.10 (0.9)	0.91 ± 0.06 (0.9)	0.94 ± 0.04 (0.96)	0.94 ± 0.04 (0.95)	0.94 ± 0.04 (0.95)	0.06	0.92	0.023
Coverage, %, mean (median)	99.3 ± 0.4 (99)	99.3 ± 0.3 (99)	99.3 ± 0.4 (99)	99.6 ± 0.3 (99.6)	99.6 ± 0.2 (99.5)	99.6 ± 0.4 (99.8)	0.19	0.54	<0.001
Beta diversity									
Bray–Curtis distance, mean (median)	0.731 ± 0.116 (0.74)	0.783 ± 0.084 (0.79)	0.825 ± 0.086 (0.84)	0.891 ± 0.119 (0.91)	0.860 ± 0.125 (0.84)	0.913 ± 0.104 (0.99)	0.01	0.01	0.001
Relative abundances of top 30 genera									
*Bacteroides*, mean (median)	0.279 ± 0.188 (0.26)	0.307 ± 0.190 (0.27)	0.253 ± 0.184 (0.22)	0.230 ± 0.166 (0.20)	0.234 ± 0.149 (0.25)	0.226 ± 0.187 (0.16)	0.22	0.71	0.01
*Faecalibacterium*, mean (median)	0.070 ± 0.052 (0.06)	0.073 ± 0.057 (0.05)	0.068 ± 0.048 (0.07)	0.072 ± 0.082 (0.04)	0.086 ± 0.088 (0.06)	0.056 ± 0.072 (0.03)	0.65	0.06	0.98
*Subdoligranulum*, mean (median)	0.061 ± 0.101 (0.03)	0.038 ± 0.054 (0.02)	0.082 ± 0.128 (0.03)	0.054 ± 0.096 (0.02)	0.034 ± 0.043 (0.01)	0.080 ± 0.133 (0.03)	0.45	0.22	0.94
*Dialister*, mean (median)	0.037 ± 0.083 (0.01)	0.044 ± 0.093 (0.01)	0.030 ± 0.073 (0.003)	0.024 ± 0.052 (0.003)	0.021 ± 0.042 (0.003)	0.027 ± 0.064 (0.002)	0.09	0.31	0.24
*Bifidobacterium*, mean (median)	0.033 ± 0.052 (0.01)	0.016 ± 0.031 (0.01)	0.049 ± 0.062 (0.02)	0.040 ± 0.071 (0.02)	0.050 ± 0.092 (0.02)	0.028 ± 0.030 (0.02)	0.09	0.71	0.21
*Escherichia shigella*, mean (median)	0.033 ± 0.071 (0.01)	0.025 ± 0.032 (0.01)	0.040 ± 0.094 (0.01)	0.048 ± 0.091 (0.01)	0.038 ± 0.093 (0.01)	0.060 ± 0.090 (0.01)	0.07	0.56	0.39
*Roseburia*, mean (median)	0.029 ± 0.055 (0.01)	0.033 ± 0.049 (0.02)	0.025 ± 0.041 (0.01)	0.013 ± 0.022 (0.01)	0.014 ± 0.019 (0.01)	0.010 ± 0.025 (0.005)	0.42	0.12	0.01
*Prevotella*, mean (median)	0.026 ± 0.078 (0.003)	0.010 ± 0.029 (0.002)	0.040 ± 0.104 (0.003)	0.035 ± 0.095 (0.002)	0.031 ± 0.103 (0.003)	0.040 ± 0.086 (0.002)	0.10	0.83	0.09
*Agathobacter*, mean (median)	0.025 ± 0.039 (0.01)	0.024 ± 0.035 (0.01)	0.027 ± 0.043 (0.01)	0.020 ± 0.035 (0.01)	0.026 ± 0.043 (0.01)	0.012 ± 0.019 (0.01)	0.95	0.28	0.41
*Parabacteroides*, mean (median)	0.021 ± 0.029 (0.01)	0.029 ± 0.035 (0.02)	0.014 ± 0.016 (0.01)	0.026 ± 0.036 (0.01)	0.030 ± 0.045 (0.01)	0.021 ± 0.021 (0.01)	0.04	0.68	0.44
*Ruminococcus gnavus* group, mean (median)	0.021 ± 0.035 (0.01)	0.017 ± 0.021 (0.01)	0.024 ± 0.05 (0.004)	0.031 ± 0.052 (0.01)	0.034 ± 0.056 (0.01)	0.027 ± 0.049 (0.01)	0.03	0.55	0.12
*Akkermansia*, mean (median)	0.021 ± 0.088 (0.001)	0.024 ± 0.105 (0.001)	0.017 ± 0.070 (0.001)	0.009 ± 0.026 (0.001)	0.010 ± 0.026 (0.004)	0.009 ± 0.027 (0.001)	0.96	0.19	0.28
*Phascolarctobacterium*, mean (median)	0.019 ± 0.029 (0.01)	0.022 ± 0.029 (0.01)	0.016 ± 0.028 (0.003)	0.022 ± 0.033 (0.01)	0.013 ± 0.017 (0.01)	0.032 ± 0.044 (0.01)	0.10	0.69	0.72
*Veillonella*, mean (median)	0.015 ± 0.028 (0.004)	0.010 ± 0.018 (0.003)	0.019 ± 0.034 (0.01)	0.013 ± 0.026 (0.003)	0.014 ± 0.025 (0.004)	0.011 ± 0.027 (0.003)	0.06	0.20	0.51
*Parasutterella*, mean (median)	0.015 ± 0.041 (0.001)	0.016 ± 0.047 (0.002)	0.013 ± 0.035 (0.001)	0.007 ± 0.019 (0.001)	0.006 ± 0.019 (0.001)	0.008 ± 0.019 (0.001)	0.82	0.73	0.21
*Fusobacterium*, mean (median)	0.013 ± 0.042 (0.003)	0.008 ± 0.013 (0.003)	0.019 ± 0.057 (0.003)	0.009 ± 0.019 (0.001)	0.012 ± 0.023 (0.002)	0.006 ± 0.012 (0.001)	0.43	0.33	0.56
*Blautia*, mean (median)	0.013 ± 0.032 (0.004)	0.020 ± 0.044 (0.01)	0.007 ± 0.012 (0.003)	0.013 ± 0.018 (0.01)	0.013 ± 0.015 (0.01)	0.012 ± 0.021 (0.01)	0.01	0.35	0.87
*Lachnoclostridium*, mean (median)	0.011 ± 0.011 (0.01)	0.014 ± 0.009 (0.01)	0.009 ± 0.012 (0.01)	0.021 ± 0.027 (0.01)	0.021 ± 0.025 (0.01)	0.021 ± 0.029 (0.009)	0.001 *	0.62	0.01
*Lachnospiraceae NK4A136* group, mean (median)	0.011 ± 0.018 (0.005)	0.013 ± 0.021 (0.01)	0.009 ± 0.013 (0.004)	0.015 ± 0.060 (0.003)	0.006 ± 0.006 (0.003)	0.025 ± 0.089 (0.003)	0.63	0.83	0.72
*Eubacterium coprostanoligenes* group, mean (median)	0.010 ± 0.025 (0.001)	0.009 ± 0.023 (0.001)	0.104 ± 0.027 (0.002)	0.018 ± 0.043 (0.003)	0.017 ± 0.038 (0.004)	0.019 ± 0.049 (0.001)	0.33	0.23	0.016
*Eubacterium eligens* group, mean (median)	0.009 ± 0.030 (0.003)	0.006 ± 0.010 (0.003)	0.013 ± 0.040 (0.003)	0.004 ± 0.010 (0.001)	0.004 ± 0.011 (0.001)	0.005 ± 0.009 (0.001)	0.76	0.83	0.16
*Alistipes*, mean (median)	0.009 ± 0.018 (0.002)	0.010 ± 0.017 (0.003)	0.008 ± 0.019 (0.002)	0.018 ± 0.040 (0.003)	0.020 ± 0.051 (0.002)	0.014 ± 0.019 (0.01)	0.26	0.44	0.10
*Haemophilus*, mean (median)	0.008 ± 0.018 (0.004)	0.004 ± 0.004 (0.003)	0.012 ± 0.024 (0.005)	0.008 ± 0.030 (0.003)	0.012 ± 0.040 (0.004)	0.003 ± 0.004 (0.002)	0.06	0.03	0.85
*Streptococcus*, mean (median)	0.008 ± 0.015 (0.003)	0.007 ± 0.014 (0.003)	0.008 ± 0.016 (0.003)	0.013 ± 0.022 (0.003)	0.013 ± 0.022 (0.003)	0.013 ± 0.023 (0.004)	0.71	0.81	0.06
*Acidaminococcus*, mean (median)	0.007 ± 0.025 (0.001)	0.011 ± 0.033 (0.001)	0.004 ± 0.012 (0.001)	0.005 ± 0.011 (0.001)	0.006 ± 0.012 (0.001)	0.005 ± 0.010 (0.001)	0.31	0.20	0.42
*Klebsiella*, mean (median)	0.007 ± 0.018 (0.002)	0.006 ± 0.013 (0.002)	0.008 ± 0.021 (0.002)	0.003 ± 0.007 (0.001)	0.002 ± 0.003 (0.001)	0.005 ± 0.010 (0.001)	0.26	0.82	0.09
*Ruminococcus*, mean (median)	0.007 ± 0.018 (0.002)	0.006 ± 0.020 (0.002)	0.008 ± 0.016 (0.002)	0.006 ± 0.012 (0.002)	0.009 ± 0.015 (0.003)	0.003 ± 0.004 (0.001)	0.09	0.04	0.56
*Collinsella*, mean (median)	0.006 ± 0.026 (0.001)	0.008 ± 0.037 (0.001)	0.004 ± 0.007 (0.001)	0.013 ± 0.037 (0.003)	0.019 ± 0.048 (0.003)	0.005 ± 0.010 (0.002)	0.97	0.14	0.35
*Ruminococcus torques* group, mean (median)	0.006 ± 0.007 (0.005)	0.006 ± 0.005 (0.005)	0.006 ± 0.008 (0.004)	0.009 ± 0.014 (0.004)	0.011 ± 0.018 (0.005)	0.005 ±0.006 (0.003)	0.21	0.13	0.24
*Flavonifractor*, mean (median)	0.006 ± 0.012 (0.003)	0.010 ± 0.016 (0.004)	0.003 ± 0.004 (0.002)	0.005 ± 0.005 (0.003)	0.005 ± 0.004 (0.003)	0.006 ± 0.006 (0.003)	<0.001 *	0.80	0.46

Data are presented as mean ± standard deviation (median). ^1^ *p*-Values were calculated using independent-samples *t*-test for continuous variables of alpha index metrics and one-way multivariate analysis of variance for continuous variables of top 30 genera. ^2^ *p*-Values were calculated using the same statistical methods between post-treatment groups. ^3^ *p*-Values were calculated using paired-samples *t*-test for continuous variables. * *p*-values were found to be significant after adjusting for multiple comparisons using the Benjamini–Hochberg procedure.

**Table 3:** Specific food frequency before and after treatment for pediatric obstructive sleep apnea.

Variable	Pre-Treatment Status (*n* = 66)	Emotional Distress		Post-Treatment Status (*n* = 53)	Emotional Distress		*p*-Value ^1^	*p*-Value ^2^	*p*-Value ^3^
		Low (*n* = 32)	High (*n* = 34)		Low (*n* = 29)	High (*n* = 24)			
Full-fat milk, scale, mean (median)	2.1 ± 1.2 (2)	2.0 ± 1.2 (2)	2.2 ± 1.2 (2)	2.6 ± 1.3 (3)	2.4 ± 1.3 (3)	2.8 ± 1.3 (3)	0.57	0.30	0.02
Low-fat milk, scale, mean (median)	0.2 ± 0.6 (0)	0.3 ± 0.7 (0)	0.2 ± 0.5 (0)	0.4 ± 0.9 (0)	0.5 ± 0.9 (0)	0.3 ± 1.0 (0)	0.62	0.67	0.23
Semi-skimmed milk, scale, mean (median)	0.02 ± 0.1 (0)	0.0 ± 0.0 (0)	0.03 ± 0.03 (0)	0.1 ± 0.3 (0)	0.1 ± 0.3 (0)	0.1 ± 0.4 (0)	0.34	0.88	0.08
Skimmed milk, scale, mean (median)	0.1 ± 0.5 (0)	0.0 ± 0.0 (0)	0.2 ± 0.7 (0)	0.1 ± 0.3 (0)	0.1 ± 0.3 (0)	0.1 ± 0.4 (0)	0.24	0.88	0.82
Orange juice, scale, mean (median)	0.7 ± 0.6 (1)	0.5 ± 0.6 (0)	0.8 ± 0.7 (1)	0.4 ± 0.6 (0)	0.3 ± 0.5 (0)	0.5 ± 0.8 (0)	0.02	0.19	0.13
Fruit drink with sugar, scale, mean (median)	1.2 ± 0.9 (1)	1.3 ± 1.0 (1)	1.2 ± 0.8 (1)	1.3 ± 0.9 (1)	1.2 ± 0.8 (1)	1.4 ± 1.0 (1)	0.45	0.41	0.82
Fruit drink without sugar, scale, mean (median)	0.5 ± 1.0 (0)	0.5 ± 0.9 (0)	0.5 ± 1.2 (0)	0.3 ± 0.7 (0)	0.2 ± 0.5 (0)	0.5 ± 0.8 (0)	0.81	0.18	0.18
Soft drinks with sugar, scale, mean (median)	0.7 ± 1.0 (0)	0.8 ± 1.0 (0)	0.8 ± 0.9 (0)	0.7 ± 1.1 (0)	0.9 ± 1.2 (0)	0.4 ± 0.8 (0)	0.76	0.08	0.70
Soft drinks without sugar, scale, mean (median)	0.3 ± 0.8 (0)	0.3 ± 0.7 (0)	0.2 ± 0.8 (0)	0.2 ± 0.5 (0)	0.2 ± 0.6 (0)	0.04 ± 0.2 (0)	0.57	0.11	0.32
Boiled potatoes, scale, mean (median)	0.3 ± 0.5 (0)	0.2 ± 0.4 (0)	0.4 ± 0.6 (0)	0.3 ± 0.5 (0)	0.2 ± 0.4 (0)	0.3 ± 0.6 (0)	0.045	0.51	>0.99
Potato chips, scale, mean (median)	0.9 ± 0.6 (1)	0.8 ± 0.7 (1)	1.0 ± 0.5 (1)	1.1 ± 0.8 (1)	1.0 ± 0.6 (1)	1.3 ± 1.0 (1)	0.22	0.09	0.02
Vegetables, scale, mean (median)	4.5 ± 1.2 (5)	4.5 ± 1.1 (5)	4.4 ± 1.3 (5)	4.5 ± 1.3 (5)	4.8 ± 1.1 (5)	4.1 ± 1.5 (5)	0.62	0.06	0.92
Fruit/berries, scale, mean (median)	3.1 ± 1.3 (3)	2.9 ± 1.5 (3)	3.3 ± 1.3 (4)	3.1 ± 1.5 (3)	3.0 ± 1.4 (3)	3.3 ± 1.6 (4)	0.31	0.56	0.85
Whole-meal bread, scale, mean (median)	0.9 ± 1.1 (1)	0.8 ± 1.1 (1)	0.9 ± 1.1 (1)	0.6 ± 0.9 (0)	0.8 ± 0.8 (1)	0.6 ± 1.0 (0)	0.63	0.48	0.28
Fish for dinner, scale, mean (median)	2.1 ± 1.6 (2)	2.2 ± 1.5 (2)	1.9 ± 1.7 (2)	2.2 ± 1.5 (2)	2.7 ± 1.5 (2)	1.7 ± 1.3 (1)	0.59	0.02	0.45
Pizza, scale, mean (median)	0.6 ± 0.6 (1)	0.4 ± 0.5 (0)	0.7 ± 0.6 (1)	0.7 ± 0.5 (1)	0.6 ± 0.5 (1)	0.8 ± 0.6 (1)	0.03	0.54	0.07
Hamburgers/hot dogs/kebabs, scale, mean (median)	0.9 ± 0.8 (1)	0.9 ± 0.7 (1)	0.9 ± 0.8 (1)	0.8 ± 0.6 (1)	0.9 ± 0.7 (1)	0.7 ± 0.6 (1)	0.97	0.28	0.73
Sweets, scale, mean (median)	1.7 ± 1.0 (2)	1.6 ± 1.1 (1)	1.9 ± 0.9 (2)	1.8 ± 1.1 (2)	1.8 ± 1.1 (2)	1.8 ± 1.1 (2)	0.20	0.81	0.64
Chocolate, scale, mean (median)	1.3 ± 0.8 (1)	1.2 ± 0.8 (1)	1.3 ± 0.9 (1)	1.2 ± 0.8 (1)	1.3 ± 0.8 (1)	1.1 ± 0.9 (1)	0.72	0.51	>0.99
Savory snacks, scale, mean (median)	1.8 ± 1.2 (2)	1.5 ± 1.2 (1)	2.2 ± 1.2 (2)	1.7 ± 1.3 (1)	1.5 ± 1.1 (1)	2.0 ± 1.4 (2)	0.03	0.15	0.92
Peanuts, scale, mean (median)	0.6 ± 0.8 (1)	0.7 ± 0.7 (1)	0.6 ± 0.8 (0)	0.5 ± 0.6 (0)	0.5 ± 0.6 (0)	0.5 ± 0.5 (0)	0.84	0.72	0.37
Cod liver oil/vitamin supplements, scale, mean (median)	0.7 ± 1.2 (0)	0.6 ± 1.1 (0)	0.8 ± 1.4 (0)	0.8 ± 1.5 (0)	0.6 ± 1.3 (0)	1.1 ± 1.7 (0)	0.46	0.27	0.12
Butter/margarine on bread (yes), % (*n*)	44 (29)	34 (11)	53 (18)	40 (21)	35 (10)	46 (11)	0.15	0.57	>0.99
Rice, scale, mean (median)	4.8 ± 1.0 (5)	5.0 ± 0.6 (5)	4.6 ± 1.2 (5)	4.7 ± 0.9 (5)	4.9 ± 0.9 (5)	4.5 ± 1.0 (5)	0.09	0.22	0.24
Noodles, scale, mean (median)	3.1 ± 1.3 (3)	2.9 ± 1.2 (3)	3.2 ± 1.4 (3)	3.0 ± 1.2 (3)	2.8 ± 1.1 (3)	3.3 ± 1.2 (4)	0.28	0.19	0.73

Data are presented as mean ± standard deviation (median) or percentage (*n*) as appropriate. ^1^ *p*-Values were calculated using independent-samples *t*-test for continuous variables of demographic variables, one-way multivariate analysis of variance for continuous variables of polysomnographic parameters and OSA-18 domain and total scores, and Fisher's exact test for categorical variables between pre-treatment groups. ^2^ *p*-Values were calculated using the same statistical methods between post-treatment groups. ^3^ *p*-Values were calculated using paired-samples *t*-test for continuous variables.

**Table 4:** Heart rate variability metrics before and after treatment for pediatric obstructive sleep apnea.

Variable	Pre-Treatment Status (*n* = 66)	Emotional Distress		Post-Treatment Status (*n* = 53)	Emotional Distress		*p*-Value ^1^	*p*-Value ^2^	*p*-Value ^3^
		Low (*n* = 32)	High (*n* = 34)		Low (*n* = 29)	High (*n* = 24)			
Total power, ms^2^, mean (median)	10,455.3 ± 7605.8 (8116)	12,569.1 ± 7804.7 (10,317)	8552.8 ± 7013.6 (5696)	7730.9 ± 5979.0 (5968)	9238.2 ± 6917.1 (6963)	5812.6 ± 3874.6 (5042)	0.045	0.04	0.002 *
ULF power, ms^2^, mean (median)	3279.2 ± 2601.3 (2316)	4006.9 ± 2864.7 (2896)	2624.3 ± 2184.7 (1794)	2462.8 ± 1751.3 (2077)	2763.1 ± 1881.3 (2149)	2080.4 ± 1527.3 (1705)	0.03	0.17	0.049
VLF power, ms^2^, mean (median)	1995.7 ± 2544.5 (1409)	2530.8 ± 3494.7 (1716)	1514.1 ± 1023.1 (1164)	1620.6 ± 1042.9 (1391)	1928.3 ± 1171.4 (1606)	1228.9 ± 697.6 (1354)	0.13	0.02	0.21
LF power, ms^2^, mean (median)	1792.6 ± 1590.8 (1217)	2216.6 ± 1725.0 (1801)	1411.1 ± 1378.7 (922)	1311.1 ± 1292.2 (940)	1602.7 ± 1535.8 (1101)	940.1 ± 778.5 (722)	0.06	0.07	0.003 *
HF power, ms^2^, mean (median)	3134.2 ± 3078.0 (1841)	3530.9 ± 2938.0 (3064)	2777.3 ± 3205.9 (1460)	2320.3 ± 3003.5 (1169)	2988.4 ± 3689.4 (1367)	1470.1 ± 1481.9 (945)	0.36	0.08	0.02 *
LF/HF ratio, mean (median)	0.811 ± 0.643 (0.56)	0.817 ± 0.580 (0.63)	0.805 ± 0.705 (0.53)	0.966 ± 0.642 (0.75)	0.924 ± 0.629 (0.75)	1.019 ± 0.668 (0.71)	0.95	0.61	0.15

Data are presented as mean ± standard deviation (median). Abbreviations: HF, high frequency; LF, low frequency; ULF, ultra-low frequency; VLF, very low frequency. ^1^ *p*-Values were calculated using independent-samples *t*-test for continuous variables of demographic variables, one-way multivariate analysis of variance for continuous variables of polysomnographic parameters and OSA-18 domain and total scores, and Fisher's exact test for categorical variables between pre-treatment groups. ^2^ *p*-Values were calculated using the same statistical methods between post-treatment groups. ^3^ *p*-Values were calculated using paired-samples *t*-test for continuous variables. * *p*-Values were found to be significant after adjusting for multiple comparisons using the Benjamini–Hochberg procedure.

**Table 5:** Multiple linear regression models of emotional distress score.

	Variable	Predictive Model of Emotional Distress			
		Regression Coefficient (95% CI)	*p*-Value	VIF	Adjusted R^2^
	Predictive model of emotional distress score				
Model 1	Shannon index	−1.6 (−2.9 to −0.2)	0.02	1.05	0.28
	*Veillonella*	31.5 (0.02 to 63.0)	0.049	1.06	
	*Bifidobacterium*	23.1 (6.7 to 39.6)	0.01	1.05	
	*Flavonifractor*	−100.3 (−171.4 to −29.3)	0.01	1.02	
Model 2	*Bifidobacterium*	41.8 (14.7 to 68.9)	0.003	1.10	0.29
	*Flavonifractor*	−256.4 (−445.3 to −67.5)	0.01	1.17	
	ULF power	−0.0004 (−0.001 to −0.00001)	0.049	1.08	
	Predictive model of change in emotional distress score				
Model 3	Change in *Veillonella*	41.3 (3.3 to 79.2)	0.03	1.02	0.14
	Change in *Agathobacter*	24.9 (2.2 to 47.6)	0.03	1.02	
Model 4	Change in *Veillonella*	47.8 (10.9–84.8)	0.01	1.02	0.20
	Change in LF power	−0.001 (−0.002–−0.0001)	0.01	1.02	

LF, low frequency; ULF, ultra-low frequency; VIF, variance inflation factor. *p*-Values were calculated using multiple linear regression models with backward selection procedures.

## Data Availability

The datasets generated and analyzed during the current study are available in the figshare repository, https://doi.org/10.6084/m9.figshare.22775159 [96].

## Abbreviations

ADHD	Attention-deficit/hyperactivity disorder
AHI	Apnea–hypopnea index
AI	Apnea index
ArI	Arousal index
ASD	Autism spectrum disorder
ASV	Amplicon sequence variant
BMI	Body mass index
ED	Emotional distress
HF	High frequency
HRV	Heart rate variability
LDA	Linear discriminant analysis
LF	Low frequency
Maaslin2	Multivariable association with linear models 2
OSA	Obstructive sleep apnea
PERMANOVA	Permutational multivariate analysis of variance
SFFQ	Short Food Frequency Questionnaire
SpO_2_	Peripheral oxygen saturation
ULF	Ultra-low frequency
VLF	Very low frequency
